# Patient patronage and perspectives of traditional bone setting at an outpatient orthopaedic clinic in Northern Tanzania

**DOI:** 10.4314/ahs.v21i1.52

**Published:** 2021-03

**Authors:** Elizabeth B Card, Joy E Obayemi, Octavian Shirima, Praveen Rajaguru, Honest Massawe, Ajay Premkumar, Neil P Sheth

**Affiliations:** 1 University of Pennsylvania Perelman School of Medicine; 2 Kilimanjaro Christian Medical Centre, Department of Orthopaedic Surgery; 3 Brown University Warren Alpert Medical School; 4 Hospital for Special Surgery, Department of Orthopaedic Surgery; 5 University of Pennsylvania, Department of Orthopaedic Surgery

**Keywords:** Bone setting, traditional medicine, traditional practitioners, orthopaedics, trauma

## Abstract

**Background:**

Much of Sub-Saharan Africa meets the rising rates of musculoskeletal injury with traditional bone setting, especially given limitations in access to allopathic orthopaedic care. Concern for the safety of bone setter practices as well as recognition of their advantages have spurred research to understand the impact of these healers on public health.

**Objectives:**

Our study investigates the role of bone setting in Tanzania through patient utilization and perspectives.

**Methods:**

We surveyed 212 patients at the outpatient orthopaedic clinic at Kilimanjaro Christian Medical Centre (KCMC) in Moshi, Tanzania. Surveys were either self-administered or physician-administered. Summary statistics were calculated using XLSTAT. Open responses were analyzed using a deductive framework method.

**Results:**

Of all surveys, 6.3% (n=13) reported utilizing traditional bone setting for their injury prior to presenting to KCMC. Of the self-administered surveys, 13.6% (n=6) reported utilizing bone setting compared to 4.3% (n=7) of the physician-administered surveys (p=0.050). Negative perceptions of bone setting were more common than positive perceptions and the main reason patients did not utilize bone setting was concern for competency (35.8%, n=67).

**Conclusion:**

Our study found lower bone setting utilization than expected considering the reliance of Tanzanians on traditional care reported in the literature. This suggests patients utilizing traditional care for musculoskeletal injury are not seeking allopathic care; therefore, collaboration with bone setters could expand allopathic access to these patients. Patients were less likely to report bone setter utilization to a physician revealing the stigma of seeking traditional care, which may present an obstacle for collaboration.

## Introduction

Prior to colonialism, traditional medicine was the sole source of health care across Africa, and it has remained the main source to this day despite growth of allopathic healthcare sectors^1^–^4^. In Tanzania, an estimated 60% of the population have their health care needs met through traditional medicine^5^,^6^. Traditional medicine encompasses a broad range of specialties including one of the most widely recognized: traditional bone setting for the care of musculoskeletal injury^7^.

In recent decades, sub-Saharan Africa has seen a rise in road traffic accidents (RTAs) and a subsequent parallel increase in the burden of musculoskeletal injury. Between 1990 and 2014, deaths due to RTAs in Sub-Saharan Africa grew by 84%^8^. Medical care for musculoskeletal injury is limited by the capacity of orthopaedic care and in many Sub-Saharan African countries traditional bone setting has helped to alleviate this growing burden^9^. Some allopathic healthcare providers have called for the formal incorporation of bone setters into allopathic healthcare by creating a referral system to increase access to standardized care^10^,^11^. However, the concern still remains regarding the safety of traditional bone setting due to complications like infection, non-union, and malunion^12^–^18^. Both viewpoints establish a need to understand the practices and patronage of traditional bone setting with the goal of training traditional bone setters to minimize adverse clinical outcomes as well as to enhance collaboration to increase access to standardized care.

Tanzania, like many countries in sub-Saharan Africa, has a severe shortage of access to orthopaedic care, with only about one orthopaedic surgeon for every 1.4 million Tanzanians^19^. However, not much is known about the role of traditional bone setting in Tanzania. To our knowledge, our study is the first to investigate Tanzanian patronage and perspectives of traditional bone setters for musculoskeletal injury.

## Methods

We surveyed patients visiting the Kilimanjaro Christian Medical Center (KCMC) outpatient orthopaedic clinic over five weeks between June and July 2017. KCMC is one of Tanzania's four large tertiary referral hospitals and serves a population of about 13 million people living in northern Tanzania. The hospital manages a majority of the orthopaedic surgical trauma cases in northern Tanzania 9.

Our survey included both multiple choice and open response questions. It was written in English, translated into Swahili by a Tanzanian translator fluent in both English and Swahili, and tested by three native Tanzanians for readability and comprehensibility. All patients who agreed to take the survey either signed or fingerprinted a consent statement and were given an information sheet in Swahili detailing the purpose and parameters of the study. Patients under 16 years of age were excluded from the study.

Surveys were either self-administered by the patient (self-administered surveys) or administered to the patient by the physician (physician-administered surveys). The self-administered method was carried out for one clinic day in June. All patients waiting before their appointment were asked to complete the written survey and 49 surveys were collected, a survey response rate of 54%. The physician-administered method was designed to include illiterate patients given a country-wide literacy rate of 78% as of 2015 20. We chose to have physicians administer the surveys after patient appointments rather than before so as to lessen belief that answers would negatively impact their care. For physician-administered surveys, providers either read the questions out loud and recorded patient responses or oversaw the patients' completion of the survey, answering any questions that arose. The sample size for the physician-administered surveys was calculated based on the expected frequency of bone setter patronage seen in the patient-administered surveys. Given the clinic sees an average of 89 patients per day, a sample size with a confidence level of 99% was found to be 80 patients over the eight days. A total of 163 physician-administered surveys were collected with 30% of total patients seen in the clinic sampled.

Survey responses were translated verbatim from Swahili into English and organized in a password protected Excel database, where breakdowns and Fisher's Exact and Chi-Square summary statistics were calculated using XLSTAT. Open responses were translated into English by a local investigator (OS) and analysis was performed by a foreign investigator (EBC) using a deductive framework method^21^.

Institutional board review was obtained from Tumaini University and Kilimanjaro Christian Medical College.

## Results

Of all patients surveyed (N = 212) the majority were male (52.5%, n = 117) and the average age was 45 years (Table 1). Most patients lived in rural areas (58.1%, n = 115) and were from the Kilimanjaro region of Tanzania (83.9%, n = 172). The primary religion represented was Christianity (77.2%, n = 159), and the primary tribe represented was Chagga (55.1%, n = 113). The highest level of education achieved by the majority of respondents was primary school (44.6%, n = 90) and most worked as farmers (32.2%, n = 55) and made under 500,000 Tanzanian Shillings (TSH), equivalent to $220 (exchange rate of 2300 TSH per 1 USD) per month (76.7%, n = 125). The most common injuries addressed at the orthopaedic clinic were to the lower extremity (60.2%, n = 165), closed fractures (39.2%, n = 82), and caused by RTAs (37.0%, n = 74) (Table 2). Approximately 50.7% (n = 104) of patients had health insurance, 95.8% (n = 69) of which was public insurance.

**Table 1:** Demographic information of surveyed patients


Gender (N = 212)	Frequency (n), Percentage

Male	117, 52.5%
Female	95, 44.8%

Age (N = 189, range = 16 – 100)	Frequency (n), Percentage

16 – 30	43, 22.8%%
31 – 45	57, 30.2%
46 – 60	51, 27.0%
61 – 75	27, 14.3%
76 – 100	11, 5.8%

Region (N = 205)	Frequency (n), Percentage

Kilimanjaro	172, 83.9%
Arusha	11, 5.4%
Tanga	8, 3.9%
Manyara	4, 2.0%
Kenya	2, 1.0%
Other	8, 3.9%

Area of Home (N = 198)	Frequency (n), Percentage

Rural	115, 58.1%
Urban	83, 41.9%

Religion (N = 206)	Frequency (n), Percentage

Christian	159, 77.2%
Muslim	38, 18.4%
Other	9, 4.4%

Tribe (N = 204)	Frequency (n), Percentage

Chagga	113, 55.1%
Pare	32, 15.6%
Sambaa	8, 3.9%
Iraq	5, 2.4%
Zigua	4, 2.0%
Other	43, 21.0%

Education (N = 201)	Frequency (n), Percentage

None	6, 3.0%
Primary School	90, 44.6%
Secondary Education	54, 26.7%
High School	7, 3.5%
Vocational School	8, 4.0%
Higher Education	37, 18.3%

Occupation (N = 171)	Frequency (n), Percentage

Farmers	55, 32.2%
Service or Sales Workers	24, 14.0%
Businesspeople	20, 11.7%
Teachers	16, 9.4%
Students	13, 7.6%
Professionals	12, 7.0%
Laborers	8, 4.7%
Retirees	7, 4.1%
Homemakers	6, 3.5%
Technicians	4, 2.3%
Armed Forces Members	3, 1.8%
Others	3, 1.8%

Monthly Income in Tanzanian Shillings (N = 163)	Frequency (n), Percentage

< 500,000	125, 76.7%
500,000 – 1,000,000	24, 14.7%
1,000,000 – 2,000,000	12, 7.4%
> 2,000,000	2, 1.2%

Self-administered and physician-administered surveys are pooled (N = 212). n = number of responses for each survey question or answer category.

**Table 2:** Location of injury or pain, diagnosis, and aetiology of injury or pain


Location of Injury or Pain (N = 270)	Frequency (n), Percentage

Lower Extremity	157, 58.1%
Upper Extremity	61, 22.6%
Back and Neck	43, 15.9%
Other	9, 3.3%

Diagnosis (N = 207)	Frequency (n), Percentage

Closed Fracture	82, 39.6%
Open Fracture	37, 17.9%
Chronic Pain	34, 16.4%
Dislocation	17, 8.2%
Ligament or Tendon Injury	11, 5.3%
Infection	2, 1.0%
Traumatic Amputation	1, 0.5%
Avascular Necrosis	1, 0.5%
Disability due to Spinal Cord Injury	1, 0.5%
Other Postoperative Complication	1, 0.5%
Unknown	20, 9.7%

Aetiology of Injury or Pain (N = 200)	Frequency (n), Percentage

Road Traffic Accident	74, 37.0%
Fall < 3m	35, 17.4%
Aging or Chronic Use	27, 13.4%
Fall > 3m	16, 8.0%
Assault	15, 7.5%
Other Trauma	8, 4.0%
Infection	3, 1.5%
Postprocedural	3, 1.5%
Undefined Bone Lesion	1, 0.5%
Unknown	18, 9.5%

Self-administered and physician-administered surveys are pooled. The “other” category for location includes head/face, abdominal, and chest injuries. Some patients presented with multiple injuries and diagnoses, but not multiple aetiologies. n = number of injuries for Location of Injury or Pain, n = number of diagnoses for Diagnosis, and n = number of aetiologies for Aetiology of Injury or Pain.

There were no differences between demographic, injury, and health insurance information between the physician- and self-administered surveys except for type and cause of injury (p < 0.05, data not shown). Patients who self-administered the surveys were less likely to report “chronic pain” and more likely to report “unknown” for type of injury. As for aetiology of injury, patients who self-administered the surveys were less likely to report “aging or chronic use”, and more likely to report “unknown”.

The prevalence of seeking care from a traditional bone setter prior to presenting to KCMC (prior-traditional bone setting (TBS) group) was 6.3% (n = 13). Of the patients who received physician-administered surveys, 4.3% (n = 7) indicated they had previously sought care from a traditional bone setter for their injury, compared to 13.6% (n = 6) from the self-administered survey group (p = 0.0500). Of all of the patients surveyed, 89.1% (n = 164) reported they would not seek care from a traditional bone setter in the future and 90.7% (n = 156) would not recommend a friend or family member to seek care from a traditional bone setter (Table 3). Patients in the prior-TBS group were not more likely to support visiting a traditional bone setter in the future or to recommend a traditional bone setter to a friend or family member (p > 0.05; data not shown). There was also no correlation between accessing care with a bone setter and income level, occupation, education, tribe, religion, gender, region, rural versus urban location, injury location, type of injury, cause of injury, insurance status, and type of insurance (p > 0.05; data not shown).

**Table 3:** Patient opinions regarding seeking future bone setting and recommending a traditional bone setter (TBS)


Seek Care for Their Injury from a TBS in the Future (N = 184)	Frequency (n), Percentage

Yes	20, 10.9%
No	164, 89.1%

Recommend a Friend or Family Member to Seek Care from a TBS (N = 172)	Frequency (n), Percentage

Yes	12, 7.0%
No	156, 90.7%
Unsure	4, 2.3%

Self-administered and physician-administered surveys are pooled. n = number of responses for each survey question.

**Figure 1:**
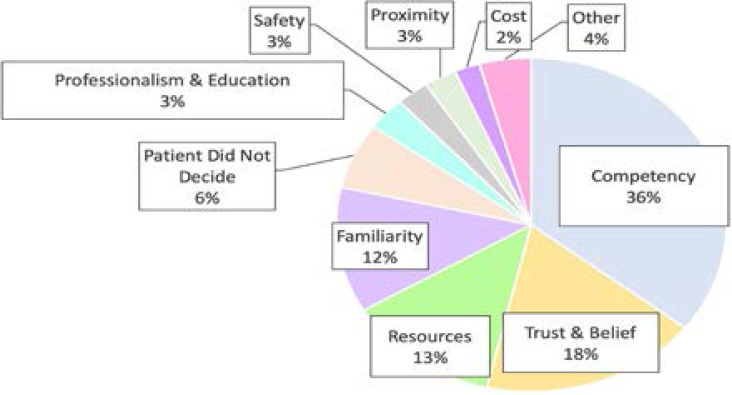
**Reasons why patients did not seek care from a traditional bone setter for their injury.** Reasons were coded into themes (n = 188).

When patients were asked to explain why they had not previously sought care from a bone setter for their injury, patients reported negative perceptions of traditional bone setters (48.4%, n = 90) more often than positive perceptions of allopathic medicine (41.4%, n = 78). The most common reasons included lack of competency of traditional providers or quality of care (35.8%, n = 67), trust or belief in care (17.6%, n = 34), equipment and resources such as diagnostic investigations (13.4%, n = 24), and familiarity with care (12.3%, n = 23) (Figure 1). Of the patients who reported competency as a reason for not seeking care from a traditional bone setter for their injury, 74.1% (n = 43) expressed a general concern for abilities and knowledge and 25.9% (n = 15) expressed concern for competency specifically regarding the diagnosis and treatment of fractures and other musculoskeletal injuries. Three patients expressed that their Christian religious beliefs were mutually exclusive with belief in traditional bone setting and two patients expressed that their education was mutually exclusive.

**Figure 2:**
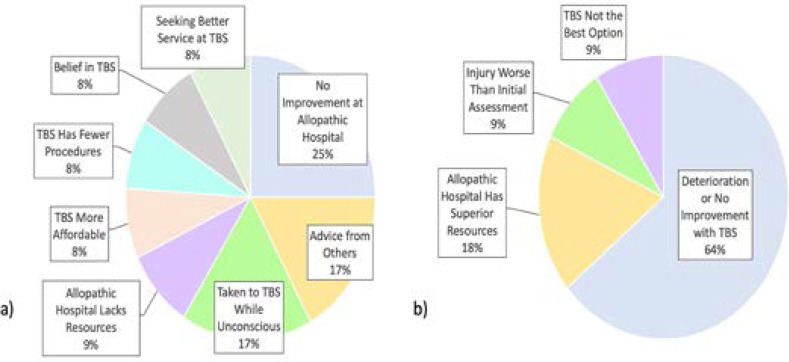
**Reasons why patients sought care from a traditional bone setter (TBS) followed by allopathic healthcare for their injury.** (a) The reasons why patients sought care from a TBS for their injury coded into themes (n = 12). (b) The reasons why patients presented to an allopathic hospital after seeking care from a TBS coded into themes (n = 11).

The main reason why patients sought care from a traditional bone setter for their injury was because their injury was not improving with allopathic medicine (25.0%, n = 3) (Figure 2). The main reason why patients then presented to an allopahic hospital was because their injury was not improving or was worsening with traditional bone setting (54.5%, n = 6).

The most common care received from a traditional bone setter was allopathic pain medicine, spiritual care, splinting, and closed fracture reduction (data not shown). According to the patients who had sought care from a traditional bone setter, 7 patients (53.8%) reported the traditional bone setters used allopathic techniques such as pain medicine, x-ray, disposable gloves, and antibiotics (data not shown).

## Discussion

We found that in the context of this tertiary referral hospital, outpatient orthopaedic clinic patients are not likely to seek care for their injury from a bone setter prior to seeking allopathic care. In other studies out of northern Tanzania, Stanifer et al. found that 56% of survey respondents from communities had used any kind of traditional medicine in the last year, while Kayombo et al. found 50% of allopathic health workers had used traditional medicine before^5^,^6^. The difference from our data showing low bone setter patronage may be because we surveyed patients about their current orthopaedic injury, a single specific ailment rather than any prior traditional medicine use as surveyed in these studies. Our results do not exclude the possibility that the surveyed patients believe traditional medicine is appropriate for conditions outside of orthopaedic conditions. Stanifer et al. and Kayombo et al. both suggested that patients in northern Tanzania commonly seek traditional medicine for systemic ailments, but fractures are much less common. Stanifer et al. identified a major determinant for use of traditional care was the “biologic understanding of disease, including its causes, symptoms, consequences, and treatment.” Participants had poor understanding of chronic diseases with symptom complexes like mental health issues, diabetes, and hypertension, which lead to more traditional medicine use for these ailments. Compared to chronic systemic disease, a lower extremity fracture after a road traffic accident has a directly visualized cause to explain symptomology^22^. This may mean better understanding of orthopaedic trauma and less utilization of traditional medicine for trauma than for chronic disease. Supportive of this, in our study some patients shared the specific belief that traditional bone setters were incompetent at treating traumatic injury while others reported allopathic providers were more competent at treating trauma.

Unlike in Tanzania, traditional bone setting in Nigeria has been well-researched with data exploring perspectives of patients regarding traditional bone setting in multiple settings. Two studies from Nigeria surveyed hospitals and found 31.6% and 17.1% of their patients with musculoskeletal injury previously patronized bone setters for their current condition, much higher than our incidence of 6.3% 23,24. Considering the setting however, these studies surveyed patients primarily in emergency departments, which likely receive a population different from those presenting to an outpatient orthopaedic clinic. For example, patients new to allopathic healthcare may end up being routed to an emergency department rather than set up with an appointment at an outpatient orthopaedic clinic.

Another potential explanation for low bone setting patronage is that patients who use traditional care are separate from patients who present to large tertiary hospitals for their care due to either geographic, financial, or cultural barriers. This study found that 83.9% of patients were from Kilimanjaro, the region in which KCMC is located, and a 2016 study out of KCMC found that for inpatient orthopaedic patients, the average travel time to KCMC was 2.3 hours^9^. This shows that the population sampled more likely came from areas with ease of access to the urban town where KCMC operates. Road infrastructure in Tanzania remains limited with only 19% of regional roads and 2% of district roads paved, which segregates large portions of the 66% of Tanzanians that live in rural areas^25^,^26^. Financially, allopathic orthopaedic care can be expensive, at times catastrophically expensive, and is more expensive than traditional care^27^,^28^. Therefore, our sample could be more financially secure than the average person within the geographic range serviced by KCMC. Furthermore, given that all surveys were collected from voluntary patients at an allopathic clinic, the population sampled likely trusts the allopathic healthcare system, which is not necessarily representative of the cultural beliefs of health care of all Tanzanians. Difference in such preferences is seen in one Nigerian report that 60% of patients in a hospital would opt for hospital care for musculoskeletal injury while for community members only 36% would opt for hospital care^29^. The majority of the prior-TBS group reported presenting to KCMC because they were not improving with traditional care suggesting that patients satisfied with traditional care do not typically present to KCMC. It is important to note that there is no data exploring the rates of traditional versus allopathic care for musculoskeletal injury in Tanzania and should be addressed in future studies.

The idea that patients who utilize traditional bone setters are not reaching large tertiary hospitals indicates need for a system of referral between traditional practitioners and allopathic providers. Like previous successes training traditional healers to triage patients, bone setters could be trained to recognize cases that require additional expertise and materials and help coordinate their referral to allopathic hospitals with the resources to treat complex musculoskeletal injuries^30^,^31^. Interestingly, over half of the patients in the prior-TBS group reported use of allopathic techniques in traditional practice, demonstrating that since bone setters in northern Tanzania are already using allopathic techniques, they may be amenable to training in these areas.

Consideration should also be given to the difference in reporting prior traditional bone setting patronage between the physician- and self-administered groups. When the patients completed the survey with a medical doctor, they were significantly less likely to report seeking care from a traditional bone setter for their injury. This finding demonstrates the potential presence of social desirability bias that led patients to underreport prior use of traditional care thereby avoiding the stigma of traditional medicine use witnessed by a medical doctor. Underreporting of preference for bone setting has been suggested previously; Nwadiaro et al. observed a discrepancy between the 74.7% of community members who reportedly preferred allopathic care despite 82.2% reporting personal or familial experience with traditional bone setters^32^.

Another demonstration of stigma was seen in the open response questions of the survey. The no-TBS group reported negative perceptions of traditional bone setting more often than positive perceptions of allopathic medicine, some of which expressed that their Christian beliefs or education were mutually exclusive with belief in traditional bone setting. The idea that western religion and education are intolerant of traditional African beliefs is not new; acceptance of western education, religion, urbanization, and globalization have all been associated with a decline in trust of traditional medicine^1^.

In the no-TBS group, the two most common themes of primary reasons for choosing allopathic care over traditional bone setting were competency and belief in the care. Similarly, one study out of Ghana found that the expertise of doctors was a large motivating factor for receiving definitive treatment at the hospital over traditional bone setting^33^. Nigerian studies report values of competence and belief supporting the opposite decision; Diamond et al. found that faith in traditional bone setting was the main motivation for seeking traditional bone setting before hospital care and Abang et al. reported belief and competence as the second and third most common reasons, respectively, the first being that others had made the decision for the patient^16^,^24^. In Sudan, Idris et al. found belief in traditional bone setting was the main motivation for choosing traditional bone setting first in both community and hospital settings^34^. In our prior-TBS group, one patient wrote that belief in traditional bone setting was their main reason for seeking a bone setter before the hospital, however, the most frequent reason was lack of improvement with allopathic care. Dissatisfaction with allopathic care is also seen elsewhere: Idris et al. found that 35.9% of patients receiving care from a bone setter had previously received care from an allopathic provider while Abang et al. reported that 46.8% of patients sought allopathic care first^16^,^34^. Interestingly, many studies mention fear as a common reason for patients to avoid seeking allopathic care, namely of amputation, metal implants, and plaster casting, and greater proportions report fear when surveying communities and bone setting facilities over hospitals^16^,^17^,^23^,^29^,^35^,^36^. Our study did not find a theme of fear in the prior-TBS group, though we cannot draw definitive conclusions given the small number in the prior-TBS group. Still given the trend in the literature in combination with our data, fear may be a motivator so powerful that patients with fear simply do not approach the hospital for care. This is important to note because fear rooted in ignorance is an identifiable target for educational initiatives. Finally, the most frequent reason for eventual presentation to the hospital after seeking traditional bone setting was lack of improvement with traditional care, which has been reported as a common motivating factor in other studies^33^,^37^.

Limitations in our study include a small number of patients in the self-administered survey group compared to the physician-administered group. The self-administered group could represent a higher literacy rate and therefore our results could potentially exclude perspectives about traditional bone setting from illiterate patients answering in the absence of a physician, though the illiterate could have employed help from family members accompanying them in the waiting room to complete the surveys. Furthermore, the physician-administered survey group was sampled at the discretion of the physicians. A cross-sectional sampling design or a randomized sampling method would have decreased sampling bias, however a cross-sectional design was not feasible in this setting given time constraints of the physicians. The two groups showed no significant differences between distributions of demographic, insurance, and injury information except for type and cause of injury. Patients who self-administered the survey were much more likely to report “unknown” for both of these questions indicating that poor health literacy and surveying prior to the clinic appointment could have been impacting patient answers.

## Conclusion

The patients at KCMC reported a much lower incidence of traditional bone setting patronage than expected considering the majority of Tanzanians meet their healthcare needs through traditional medicine. This suggests that either patients tend to avoid traditional medicine for orthopaedic injuries specifically, or that those accessing traditional care for musculoskeletal injury are not seeking care at KCMC. In the latter scenario, collaboration with bone setters through a referral system could expand allopathic care access to these patients. Patients were less likely to report bone setting patronage to a physician, which underscores the stigma of seeking traditional care in an allopathic setting. The reservations of society to see traditional healing gain societal relevance provide an obstacle to successful collaboration and integration among allopathic and traditional providers^38^. Efforts must be made to narrow the gap between these two treatment approaches in order to grant access to a majority of patients that require musculoskeletal care.
